# Editorial: Alternative Splicing Regulation in Plants

**DOI:** 10.3389/fpls.2020.00913

**Published:** 2020-07-09

**Authors:** Ezequiel Petrillo, Maria Kalyna, Kranthi K. Mandadi, Shih-Long Tu, Craig G. Simpson

**Affiliations:** ^1^Departamento de Fisiología, Biología Molecular y Celular, Facultad de Ciencias Exactas y Naturales, Universidad de Buenos Aires, Buenos Aires, Argentina; ^2^CONICET-Universidad de Buenos Aires, Instituto de Fisiología, Biología Molecular y Neurociencias, Buenos Aires, Argentina; ^3^Department of Applied Genetics and Cell Biology, BOKU—University of Natural Resources and Life Sciences, Vienna, Austria; ^4^Texas A&M AgriLife Research and Extension Center, Texas A&M University, Weslaco, TX, United States; ^5^Department of Plant Pathology and Microbiology, Texas A&M University, College Station, TX, United States; ^6^Institute of Plant and Microbial Biology, Academia Sinica, Taipei, Taiwan; ^7^Cell and Molecular Sciences, James Hutton Institute, Dundee, United Kingdom

**Keywords:** splicing factor, development, stress, adaptation, evolution, environment, flowering, germination

Research on alternative splicing (AS) in plants has bloomed during the past decade, largely fueled by the advance of high-throughput sequencing (HTS) technologies and pioneering papers demonstrating an unexpectedly high frequency of AS in plants (Filichkin et al., 2010; Lu et al., 2010; Marquez et al., 2012; Syed et al., 2012). The formation and regulation of alternative transcripts from individual transcribed genes by alternative splice site selection pervades all aspects of a eukaryote's development and adaptive response to its changing environment. This is particularly relevant to sessile plant species that must be able to rapidly respond to abiotic, biotic, diurnal, and seasonal fluctuations. The mechanism of pre-mRNA splicing and the process of splice site selection has existed since its divergence from metazoans (Fedorov et al., 2002) and is regulated by splicing factors that are components of the assembling spliceosome. Many knowledge gaps remain to be addressed, not only to define the AS prevalence in different plant species and its impact on various biological processes, but also to understand its mechanistic basis with the aim of manipulating crops for important traits required for food security. Here, we share with the plant biology community a Research Topic that aims to showcase current findings, emerging questions, and technical advances, in the field of AS in plants.

The basic mechanisms underlying AS regulation in different eukaryotes are quite conserved. Core sequence elements are found at the 5′ splice site, 3′ splice site and branchpoint located upstream of the 3′ splice site (Wahl et al., 2009; Meyer et al., 2015). Splicing enhancers and silencers positioned in introns and exons further define selected splice sites. These sequence elements are relatively short and variable such that multiple alternative sequences exist and, along with variability in the expression of splicing factors, lead to AS. However, there are splicing differences between plants and animals that highlight peculiarities in gene and chromatin architecture, transcription, and splicing machineries. Chaudhary et al. review differences in AS mechanisms and its regulation in plants and humans. In animals, it is widely accepted that splicing and transcription are coupled, and emerging evidence indicates that it is conserved in plants (Chaudhary et al.). The co-transcriptional behavior of splicing means that the chromatin environment and the RNA Polymerase II (RNAPII) processivity have a strong influence on splicing outcomes. In plants, light/dark transitions modulate the RNAPII elongation rate, which in turn controls AS, demonstrating a coordination between AS, transcription and plant growth (Godoy Herz and Kornblihtt). Cao and Ma summarized the role of a highly conserved SKI-INTERACTING PROTEIN (SKIP), which functions both as a transcription factor and as a general splicing factor. It is not only required for precise and efficient splicing of pre-mRNAs on a genome-wide scale, but also delays flowering time by promoting transcription of the flowering repressor *FLC* (Cao and Ma). SKIP also interacts with ELF7, an RNAPII-associated factor 1 complex (Paf1c) component, that regulates transcription elongation. Cao and Ma further outlined SR45 (RNPS1 human ortholog) as a multifunctional splicing factor, while Chen et al. described a proteomic study of the *sr45-1* mutant that identified this factor as a component of the apoptosis and splicing-associated protein complex (ASAP), which is known to regulate RNA metabolism at multiple levels by recruiting histone deacetylases to the *FLC* locus. The *sr45-1* mutant also showed a significant reduction in Sin3-associated protein 18 (SAP18), a component of ASAP, which induces transcriptional silencing in mammalian cells. Lastly, yeast PRE-mRNA-PROCESSING PROTEIN 40 (PRP40) has a role in the early steps of spliceosome complex formation, and the human homolog was initially discovered as a transcriptional modulator and later linked to pre-mRNA splicing. Hernando et al. characterized a mutant of the *Arabidopsis* PRP40C, and found the factor linked the regulation of gene expression and pre-mRNA splicing to modulate plant growth, development, and stress responses in *Arabidopsis*. These multifunctional splicing factors show the complex interplay between splicing and transcription that underlie functional coupling between these processes.

Plants adapt to changing conditions and acquire tolerances to daily, seasonal, and chronic stresses. Proteomics of *sr45-1* revealed increased amounts of enzymes involved in glucosinolate biosynthesis, which are important for disease resistance (Chen et al.). Nimeth et al. uncovered the hidden potential of AS in the DNA damage response in plants by reporting that about 80% of the DNA repair genes are alternatively spliced based on the evidence in the *Arabidopsis* reference transcript dataset (AtRTD2). Hernando et al. found contrasting sensitivity of the PRP40C mutants to salt stress and their enhanced tolerance to *Pseudomonas* infection. They identified over 600 transcripts enriched for genes related to biotic and abiotic stress responses. In both cases, increased proportions of intron retention events were identified, indicating a possible mechanism for regulating expression of stress response genes and a role in fine tuning transcriptome functionality (Hernando et al.). Many intron retention transcripts are constrained in the nucleus avoiding degradation by nonsense-mediated mRNA decay. Some may be translated to produce truncated proteins that could modulate the function of the fully spliced protein (Chaudhary et al.). Precise control of plant developmental transitions, ranging from seed germination to flowering, is essential for plant propagation and reproductive success. Many transitions are initiated in response to changes in temperature and light. For instance, light perception plays key roles in the transition from seed dormancy to germination that involves red and far-red photoreceptors. Tognacca et al. showed that a pulse of red light changes AS of several genes, mostly involved in splicing regulation, light signaling or dormancy/germination, supporting an important role of AS at germination. Flowering time is regulated by a complex network of factors that integrate environmental and developmental cues. Park et al. reviewed the established roles of alternatively spliced genes that are essential for flowering time. For example, alternatively spliced *CONSTANS* (*CO*) produces COα and COβ protein isoforms, which form non-DNA-binding heterodimers during photoperiodic flowering and protein turnover (Park et al.). Alternatively spliced *FLOWERING LOCUS M* (*FLM*) encodes a temperature sensitive, MADS box transcription factor functioning as a floral repressor. The protein isoform FLM-β is the functional floral repressor, while FLM-δ has a reduced DNA-binding capability and inhibits FLM-β. Nibau et al. showed that the cyclin-dependent kinase G2 (CDKG2), together with its cognate cyclin, CYCLIN L1 (CYCL1) affects the AS of *FLM*, balancing the levels of the FLM-β and FLM-δ isoforms across a temperature range. Thermosensory and developmental signal induction of AS is therefore important for fine-tuning the initiation of flowering. These genes produce apparently functional protein isoforms translated from alternatively spliced transcripts. However, the full extent to which alternatively spliced transcripts are translated and contribute to protein diversity is still far from clear. COα and COβ protein levels change during the day but alternative transcript levels remain constant. A significant reduction in SAP18 protein with no significant decrease in *SAP18* RNA is observed in the *sr45-1* mutant (Chen et al.). There is a poor correlation between alternatively spliced transcripts and detectable proteins from proteomic experiments. How this disparity occurs remains to be determined. It is possible that the transcript abundances, transcript stability, transcript retention in the nucleus and other processes will ultimately impact on the proteomic outputs. Mis-interpretation of transcript translation by disregarding authentic start codons and premature termination codons may also lead to false interpretation of proteomic outputs. Alternatively, the proteomic technologies, as opposed to PCR or HTS approaches, may not be sensitive enough to detect the low-abundant proteins arising from AS variants (Brown et al., 2015; Zhang et al., 2017).

Genetic variability at core splicing elements, splicing regulatory sequences or in genes encoding trans-acting splicing factors can substantially affect the abundance of transcript isoforms. These changes impact phenotypic diversity and terrestrial adaptation. Khokhar et al. used 666 diverse natural inbred *Arabidopsis* ecotypes, demographically sourced along the east-west axis of Eurasia. They performed splicing quantitative trait loci (sQTL) analysis to reveal the functional impact of genetic variations on AS patterns of genes related to stress response, flowering and the circadian clock (Khokhar et al.). Dantas et al. analyzed the AS events of circadian clock genes in the C4 sugarcane under different field conditions. The authors found striking differences in the seasonal AS patterns in these genes that could be a result of fluctuating temperatures between winter and summer (Dantas et al.). The strong association between AS and environmental stimuli, including temperature, diurnal, and seasonal changes, underscores that AS is intricately involved in myriad of adaptation processes. SR proteins are a highly conserved family of AS regulators. Melo et al. performed an *in silico* analysis that identified 16 *Physcomitrella patens* SR genes belonging to the six canonical plant SR protein subfamilies. The number and size of SR subfamilies changes greatly from aquatic green algae to vascular plants. The authors suggest a role for SR proteins in the adaptation to new land habitats and perhaps even speciation (Melo et al.). Ling et al. discuss the evolution of AS in plants compared to that in vertebrates. They performed a comparative analysis of the transcriptomes of both closely and distantly related plants and found a low level of AS conservation among different species with the exception of AS events that generate premature termination codons (Ling et al.). Clark et al. analyzed genome-wide AS in tomato by integrating mRNA, EST, and RNA-seq reads from 27 published projects. They found an ~65% frequency of AS, similar to the frequency detected for *Arabidopsis* (Clark et al.). Lastly, Bedre et al. discuss the value, limitations and future developments of HTS technologies needed to overcome limitations imposed by low coverage of particular genomes, high ploidy levels and sequencing error rates (Bedre et al.). Melo et al. further bolster these conclusions by identifying discrepancies in major databases when characterizing SR genes in *Physcomitrella* that resulted from imperfect gene annotation curation, sometimes lacking support by expression data (Melo et al.). Nevertheless, new HTS and bioinformatics approaches will likely spur further in-depth identification of AS patterns on a genome-wide scale in diverse plant species that coupled with the fundamental mechanistic studies will likely answer key questions related to the role of AS in adaptation and evolution of plants on this planet.

## Author Contributions

All authors listed have made a substantial, direct and intellectual contribution to the work, and approved it for publication.

## Conflict of Interest

The authors declare that the research was conducted in the absence of any commercial or financial relationships that could be construed as a potential conflict of interest.
